# Computer-assisted resilience training to prepare healthcare workers for pandemic influenza: a randomized trial of the optimal dose of training

**DOI:** 10.1186/1472-6963-10-72

**Published:** 2010-03-22

**Authors:** Robert G Maunder, William J Lancee, Reet Mae, Leslie Vincent, Nathalie Peladeau, Mary Agnes Beduz, Jonathan J Hunter, Molyn Leszcz

**Affiliations:** 1 Mount Sinai Hospital, Toronto, Ontario, Canada; 2 Department of Psychiatry, University of Toronto, Ontario, Canada; 3 Faculty of Nursing, University of Toronto, Ontario, Canada

## Abstract

**Background:**

Working in a hospital during an extraordinary infectious disease outbreak can cause significant stress and contribute to healthcare workers choosing to reduce patient contact. Psychological training of healthcare workers prior to an influenza pandemic may reduce stress-related absenteeism, however, established training methods that change behavior and attitudes are too resource-intensive for widespread use. This study tests the feasibility and effectiveness of a less expensive alternative - an interactive, computer-assisted training course designed to build resilience to the stresses of working during a pandemic.

**Methods:**

A "dose-finding" study compared pre-post changes in three different durations of training. We measured variables that are likely to mediate stress-responses in a pandemic before and after training: confidence in support and training, pandemic-related self-efficacy, coping style and interpersonal problems.

**Results:**

158 hospital workers took the course and were randomly assigned to the short (7 sessions, median cumulative duration 111 minutes), medium (12 sessions, 158 minutes) or long (17 sessions, 223 minutes) version. Using an intention-to-treat analysis, the course was associated with significant improvements in confidence in support and training, pandemic self-efficacy and interpersonal problems. Participants who under-utilized coping via problem-solving or seeking support or over-utilized escape-avoidance experienced improved coping. Comparison of doses showed improved interpersonal problems in the medium and long course but not in the short course. There was a trend towards higher drop-out rates with longer duration of training.

**Conclusions:**

Computer-assisted resilience training in healthcare workers appears to be of significant benefit and merits further study under pandemic conditions. Comparing three "doses" of the course suggested that the medium course was optimal.

## Background

This report concerns pilot testing of a computer-assisted training course which was intended to build resilience to stress in healthcare workers facing an influenza pandemic. In particular, this study aimed to determine the optimal "dose" of such training by comparing three versions of the course (differing in duration) on pre-post changes in variables that were considered proximal determinants of resilience.

Well before the onset of the H1N1 influenza pandemic in 2009, pandemic preparedness plans recognized that psychological support of healthcare workers would be necessary during an influenza pandemic [1-4]. Observations made during and after the 2003 outbreak of severe acute respiratory syndrome (SARS) suggested that an emerging infectious disease causes stress in healthcare settings because of fear of contagion [5-7], concern for family health [6,8,9], job stress [6,7], interpersonal isolation [6,7], quarantine [10] and perceived stigma [7,10,11].

Moving beyond plans to support affected healthcare workers after exposure to a pandemic, evidence suggests that psychosocial support and training should be provided to healthcare workers prior to a pandemic to build resilience [12], thereby reducing the impact of stress after exposure. This may be important because predictive models often assume high absenteeism due to illness in healthcare workers and their families, and the stress of dealing with an extraordinary outbreak of infectious disease may further increase absenteeism [13]. Stress-related absenteeism, which may be avoidable with intervention, provides a "second-hit" on a healthcare system which is compromised by other unavoidable losses of human resources. The importance of discretionary absenteeism was highlighted by a report that up to 53% of healthcare workers would refuse to attend work if multiple patients infected with pandemic influenza were admitted to their hospital [14].

We designed an educational intervention to improve resilience to pandemic-related stress. Resilience has been defined as overcoming stress or adversity or, more precisely, as having a good outcome after an adverse experience [15]. The resilience literature is large and varied [16], ranging from studies of individual differences between children that promote healthy outcomes after early life adversity [15] to the factors that protect individuals exposed to extreme traumatic stressors [17,18] or protect adults from mental illness [19]. Nurses' resilience to workplace stresses may be bolstered by processes that enhance the quality of professional relationships and that foster the development of emotional insight, life balance, spirituality, and reflective thinking [20]. Our intention was to design and implement a training intervention which healthcare workers could take prior to an influenza pandemic that would reduce the adverse stress-related effects of a subsequent pandemic exposure.

We first considered the educational goals. Outcomes of pandemic-related resilience, such as reduced event-related absenteeism and psychological stress, cannot be measured until exposure to a pandemic occurs. For this pilot we focused instead on proximal outcomes which could facilitate resilience. Studies of the individual and health-system variables which mediated the outcomes of stress related to SARS suggested that the best goals of training would be to (i) increase confidence in being well-supported by the hospital and well-prepared for the pandemic and (ii) enhance adaptive strategies of coping (increasing problem solving and seeking support and decreasing escape-avoidance) [13]. Since the purpose of effective training is to change the behavior of healthcare workers in a pandemic situation, we also proposed, based on the theory of social learning [21], that enhancing self-efficacy (expectations about personal ability to respond adaptively to pandemic-related stresses) should be a goal of training because it is expected to be a proximal predictor of behavior [22]. Finally, since many stressful aspects of an infectious disease outbreak are directly or indirectly of an interpersonal nature (e.g. concern for family health, interpersonal job stresses, interpersonal isolation and perceived stigma) and because interpersonal problems are associated with job stress in healthcare workers [23], reducing interpersonal problems was a goal. These pandemic related stressors could be expected to amplify the stress and strain which is commonly experienced by health care workers in the contemporary health care environment [24].

We next considered the optimal format of training. Since hundreds of thousands of healthcare workers around the world are affected by an influenza pandemic, resilience training should be available at reasonable cost on a very large scale. This is a challenge. Those modes of continuing professional education that demonstrate strong effectiveness regarding behavioral change are resource-intensive interventions designed for the training of individuals and small groups [25]. On the other hand, computer-assisted and Internet-based learning technologies have been promoted as effective strategies to facilitate learning, which may overcome barriers related to timing and cost [26]. Computer-assisted learning also has the advantages of standardization of the course material for all learners and enhanced opportunities for personalization of timing, pace and opportunities for review. It can be provided to large and widely distributed groups of people and, beyond the costs of developing the course, the incremental cost per learner may be modest.

Despite a growing body of literature on computer-assisted and Internet-based continuing professional education for health professionals, most studies are methodologically weak [27,28]. Computer-assisted learning has been shown to be a feasible option for educating professionals when compared with printed and/or lecture based continuing education [29]. Compared to no intervention, Internet-based training has a large effect on learner satisfaction, acquisition of knowledge, skills and behavioral change [28]. Compared to other learning modalities the effects on these outcomes have been small, non-significant and inconsistent [28,30-32]. Thus, there is no strong evidence base for the superiority of any particular modality. The application of computer-assisted learning to the goals and context of pandemic preparation is untested. Thus one purpose of our research study was to determine if the goals of pandemic resilience training could be accomplished with a computer-assisted educational course that could be widely distributed and self-administered.

Finally, we considered the content of the course and the types of learning experience that might achieve the identified goals. This consideration was guided by experience counseling healthcare workers during and after SARS [9], and by knowledge from other learning resources. We assumed that information about influenza and about stress and coping should be provided but would not, in itself, achieve the goals of training. Based on research in continuing professional education, we expected that the format of the course should address both objective and perceived learning needs, and include both cognitive interactivity and practicing of skills [25,33]. The effect of these modes of training on educational outcomes of Internet-based training has been inconsistent, although practice of prescribed exercises more consistently contributes to skills acquisition [28]. Based on the experience of counseling workers during and after exposure to SARS, we expected that exercises should be provided that not only involve cognitive interactivity but also exposure to affectively charged interpersonal events. The goal of these exercises is to enhance reflective, as opposed to immediately reactive responses to acute interpersonal stressors. The principle of increasing reflection had been valued by our colleagues during facilitated group discussion of SARS-related stressors [34]. We also drew upon previous work on psychological first aid [35,36], stress management [37], and coping [38].

The resulting course was named the Pandemic Influenza Stress Vaccine. We hypothesized that the course would lead to improvements in satisfaction with support and training, coping, pandemic-related self-efficacy and interpersonal problems. It was not known at the time of developing the course what the ideal "dose" of this intervention would be, in terms of the time spent on the course by learners and the comprehensiveness of its curriculum. Previous studies of Internet-based learning have shown that longer duration is positively associated with behavioural outcomes [28]. We expected that a course that was too brief might not achieve the teaching goals while a course that was too long might be unnecessarily burdensome and lead to dropping out. The purpose of this study was to test three versions of the course (short, medium and long) with respect to improvements at the end of the course in the hypothesized outcomes and drop-out rates.

## Methods

The setting for this study was Mount Sinai Hospital, a teaching hospital in Toronto, Canada which was directly affected by the 2003 SARS outbreak. All employees and professional staff at were eligible to participate in the study, which was conducted from September 2008 to January 2009. Information about the study was presented at staff meetings and rounds in departments throughout the hospital. Employees and staff who consented to participate were randomized to one of three course lengths: short, medium or long. In order to reduce technological challenges, for the sake of this pilot study the course was accessed on a computer flash drive rather than via the Internet. Participants were provided with a computer flash drive containing the course and instructions for self-administering the training. Participants were instructed to complete the course in several sittings at their own pace, working alone in a quiet setting that allowed them to focus on learning. Participants used the course on computers connected to the Internet, which allowed information provided by the participants (when answering questions or providing information in interactive exercises) to be transmitted back to the researchers and entered in the study database. This process allowed researchers to monitor the progress of participants through the course and the timing of completion. Neither the research assistant administering the course, nor the participant knew which course length was assigned. However, assignment could not be truly blinded because participants were informed that different lengths of the course were being compared and that the typical cumulative duration of these courses was 1.75 hr, 3 hr and 4.5 hr respectively. Those who completed the course received CPE credit. Except for physicians, course completers also received paid educational time. Staff (except physicians) who were not usually entitled to paid educational time were reimbursed at the same rate as staff nurses. The study was approved by the Mount Sinai Hospital Research Ethics Board.

### The Pandemic Influenza Stress Vaccine

The course consisted of modules incorporating different modalities of learning. Knowledge-based modules addressed the topics listed below using audio and video mini-lectures accompanied by onscreen notes and printed fact sheets. Quizzes and games provided brief diversions and reinforced knowledge. Relaxation skills were taught with audio modules guiding participants in progressive muscle relaxation, relaxation breathing, imagery, and combined techniques. Self-assessment modules used psychological questionnaires to characterize interpersonal problem and coping style. Feedback was provided that was both individualized and relevant to the context.

### List of topics addressed in the Pandemic Stress Vaccine

What to expect during a pandemic.

What is resilience?

Normal stress responses.

How to help others with stress (psychological first aid).

Working outside your comfort zone.

Moral and ethical dilemmas.

An approach to coping.

Active listening.

Expressing yourself constructively.

Balancing family and work.

How to talk to children about disasters and emergencies.

Personal and home preparation.

Managing drugs and alcohol.

Danger signals & resources for getting help.

Interactive reflective exercises consisted of four parts. First, participants watched a 1-3 minute video dramatization of a pandemic-related scenario designed to be realistic and stressful. For example, in one video dramatization (Additional file 1) a nurse is confronted in the hallway by a patient's family member who demands to know why her family member is not receiving a scarce medication and is clearly not satisfied with the nurse's reply. Participants are asked to imagine themselves in a character's role while they watch. Second, participants described responses to the scenario by completing the stems of 3 sentences: "On a good day I might...", "On a bad day I might..." and "Somebody else might..." Third, participants reflected on their immediate response to the scenario and considered a highly individualized menu of explanations for their response, alternative responses and constructive next steps. There are more than 3000 possible logical paths through this exercise, but a sense of the experience can be conveyed with an example. We illustrate the first few steps of one possible path through reflecting on the scenario described above.

Q: What is your initial reaction? A: It makes me angry.

Q: In what way does this make you angry? A: I deserve more respect.

Q: You have answered, "I deserve more respect". How can you make this difficult situation work? A: I will be clear and open in communicating with others.

Q: You recognize the importance of clear communication in this situation. What can you do to facilitate effective communication? Etc. (the reflective exercise continues).

The menu items available for responses, and thus the possible paths through the exercise, are specific to the context of each scenario. Fourth, the participant receives feedback in the form of a brief narrative description of their "reflective path." Each reflective exercise can be completed in less than 5 minutes.

### Instruments

Confidence in training and support was measured with a questionnaire derived to measure responses of healthcare workers to SARS [39] that was found to predict long-term effects of SARS [13], modified to apply to influenza. Nine items (e.g. "If I have problems using equipment in an influenza pandemic, I am confident that I will have someone to ask for help") are rated on a 5-point scale from 1 (strongly disagree) to 5 (strongly agree). Summed item scores were normally distributed. Cronbach's alpha was 0.85.

Perceived efficacy to adapt to pandemic conditions was measured with the Pandemic Self-Efficacy Scale, authored for this study. This 24-item scale measures attitudes toward working in a pandemic (e.g. "How confident are you now that in the event of an influenza pandemic you will be able to do your job effectively, even if you are stressed or tired?") rated on a 5-point scale from 1 (Not confident at all. I don't think I can do this) to 5 (Very confident. I am sure of it). Scores were normally distributed. Cronbach's alpha was 0.93.

Interpersonal problems were measured with the 32-item Inventory of Interpersonal Problems (IIP-32), an abbreviated version of the IIP-64. Participants rate the degree to which they experience interpersonal problems on a 5-point scale from 0 (not at all) to 4 (extremely). Each of the eight subscales of the IIP (Controlling, Self-Centered, Cold/Distant, Socially Inhibited, Nonassertive, Overly Accommodating, Self-Sacrificing, and Intrusive/Needy) are calculated as the mean of 4 items. The IIP has adequate test-retest reliability, convergence to an established measure of interpersonal styles, and responsiveness to changes in psychotherapy [40,41]. Total interpersonal problems (sum of 32 item scores) were distributed as a truncated normal curve (skewed toward 0, range 6 - 75). Cronbach's alpha was 0.90.

Coping via problem-solving, seeking support from others and escape-avoidance were measured with subscales of the Ways of Coping Inventory [42,43] a widely used instrument which yields eight subscales of coping strategies, which are supported in clinical and non-clinical samples [44]. In this study coping scales were selected that have predictive power with respect to long-term stress-related outcomes of working during the SARS outbreak [13]. Coping scales were calculated as the mean of item scores on a 4 point scale from 0 ("Not used") to 4 ("Used a great deal"). The reliability of the subscales has been adequate in many studies (typically 0.60 to 0.75) [45]. Problem-solving and seeking support were both normally distributed. Cronbach's alpha was 0.76 for problem-solving and 0.77 for seeking support. Escape-avoidance was skewed toward zero. Cronbach's alpha 0.73.

### Analysis

Drop-out rates were calculated as proportions and between-group differences in proportions were tested with Pearson's chi-square. An intention to treat analysis of outcome variables was used comparing pre-course (T1) and post-course (T2) values of outcome variables using paired T-tests for all subjects who started the course. Subjects who dropped out between T1 and T2 were assigned T1 values of outcome variables at T2 (i.e. were assigned a pre-post change of zero). Pre-post differences were expressed as mean and 95% confidence intervals of means.

## Results

**Figure 1:**
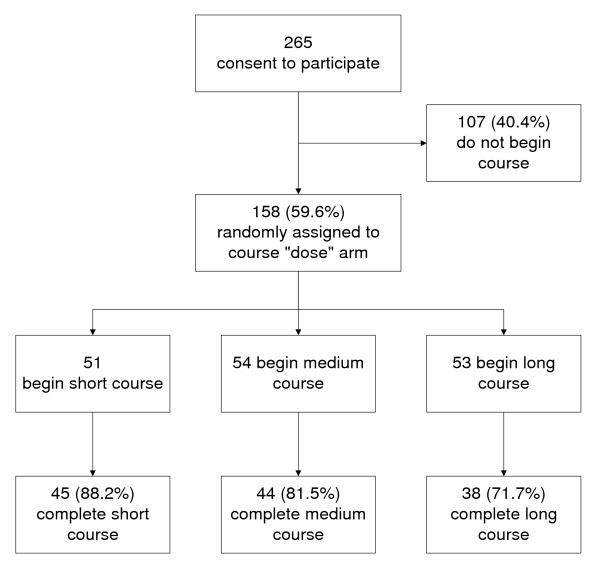
**Recruitment and participation flowchart**.

Most participants (86%) were women and more than half (54%) were nurses (Table 1). Of 265 healthcare workers who consented to participate, 158 (59.6%) started the course (recruitment and retention flowchart, Figure 1). The proportion of people dropping out, either before or after starting the course, differed by job type and gender (Table 1). Among nurses, 58% who consented to participate started the course (84 of 144) compared to 87% (45 of 52) of other professionals and 42% (29 of 69) of non-professional staff. Women were more likely to start the course (62%) than men (44%).

**Table 1:** Distribution of course completers and drop-outs by job type and gender.

	Post-consent drop out (did not start)		Mid-course drop out (did not finish)		Completed course		Total	
	**n**	**%**	**n**	**%**	**n**	**%**	**n**	**%**

**Job type**								

Health professional - nurse	60	56%	18	58%	66	52%	144	54%

Health professional - other	7	7%	6	19%	39	31%	52	20%

Other staff	40	37%	7	23%	22	17%	69	26%

**Gender**								

Female	87	81%	25	81%	117	92%	229	86%

Male	20	19%	6	19%	10	8%	36	14%

**Total**	107		31		127		265	
Difference in drop-out status by job type, Chi-square = 26.7, df = 4, p < 0.001								
Difference in drop-out status by gender, Chi-square = 6.8, df = 2, p = 0.03								

Of the 158 hospital workers who started the course, 127 (80%) completed it. There was no significant difference between mid-course drop-outs and course completers with respect to gender or job type. Failure to complete the course after starting occurred in 6 of 51 participants (11.8%) assigned to the short course, 10 of 54 participants (18.5%) assigned to the medium course and 15 of 53 participants (28.3%) assigned to the long course. The trend to greater drop-outs with longer course was nonsignificant (Chi-square = 4.6, df = 2, p = 0.10). The cumulative time required to complete the course is illustrated in Table 2.

**Table 2:** Total (cumulative) duration of the Pandemic Stress Vaccine course.

	Number of sessions assigned	Cumulative time spent on course (min)		
		**Minimum**	**Maximum**	**Median**

Short course	7	55	173	111

Medium course	12	75	362	158

Long course	17	124	414	223

Participants showed significant improvements from the start to the end of the course in pandemic self-efficacy, confidence in training and support and interpersonal problems (Table 3). Dimensions of interpersonal problems which improved included problems with being socially inhibited, nonassertive, overly accommodating, self-sacrificing and intrusive/needy. Dimensions of interpersonal problems which did not improve were being controlling, self-centered and cold/distant (Table 3). It is noteworthy that the severity of interpersonal problems at T1 was mild, with mean scores near a score of 1 on a 0 to 4 scale. For the dimensions of interpersonal problems which did not show a significant overall change, the median score at T1 was 0.25 to 0.5. We therefore tested if there was a change in these dimensions among participants who reported problems (score > 0) at T1. Among these participants there was a significant pre-post decrease in problems with being cold-distancing (n = 104, pre: 0.9 ± SD 0.8; post 0.8 ± 0.8; 95% CI of difference -0.2 - 0.0, p = 0.02) and self-centered (n = 99, pre: 1.0 ± SD 0.9; post 0.8 ± 0.9; 95% CI of difference -0.4 - -0.1, p = 0.001) but no significant change in controlling behavior.

**Table 3:** Changes in outcome variables from start to finish of course.

					Pre-Post Difference^1^			
					
	Start of Course		End of Course		95% Confidence Interval			
**Variable**	**Mean**	**SD**	**Mean**	**SD**	**Mean**	**Lower**	**Upper**	**P**

Pandemic self-efficacy	87.7	12.6	92.9	12.9	5.1	3.7	6.6	<0.001
								
Confidence in support and training	32.6	4.9	33.8	4.7	1.1	0.7	1.6	<0.001
								
Interpersonal problems								
Controlling^2^	0.4	0.5	0.4	0.5	0.0	-0.1	0.0	0.55
Self-centered^2^	0.7	0.9	0.5	0.8	-0.1	-0.2	0.0	0.01
Cold-distant^2^	0.6	0.8	0.6	0.6	-0.1	-0.2	0.0	0.10
Socially inhibited^2^	1.0	0.9	0.9	0.8	-0.2	-0.1	0.0	0.001
Nonassertive^2^	1.4	0.9	1.3	0.8	-0.2	-0.3	-0.1	0.001
Overly accommodating^2^	1.6	0.9	1.4	0.8	-0.2	-0.2	-0.1	<0.001
Self-sacrificing^2^	1.4	0.8	1.2	0.8	-0.2	-0.2	-0.1	<0.001
Intrusive-needy^2^	0.8	0.7	0.7	0.8	-0.1	-0.2	0.0	0.003
Total problems^3^	31.4	16.0	27.6	15.6	-3.7	-5.1	-2.3	<0.001
								
Ways of Coping								
Problem-solving	1.5	0.7	1.5	0.7	0.0	-0.1	0.0	0.95
Seeking support	1.5	0.7	1.4	0.6	0.0	-0.1	0.0	0.40
Escape-avoidance	0.6	0.5	0.6	0.5	-0.1	-0.1	0.0	0.06

^1 ^Significance of pre-post differences by paired t-tests, based on intention to treat.

^2 ^Mean score of 4 items scored 0 to 4.

^3 ^Summed score of 32 items scored 0 to 4.

Coping with stress using problem-solving, seeking support from others or through escape-avoidance did not change over the course (Table 3). Since coping with problem-solving and seeking support was common at the start of the course, we tested if there was a pre-post improvement among people who were using less of these strategies (score <1.5). Among those who were under-utilizing problem-solving at T1, there was a significant pre-post increase (n = 72, pre: 0.9 ± SD 0.9; post 1.2 ± 0.6; 95% CI of difference 0.2 - 0.4, p < 0.001). Similarly, among those who were under-utilizing support-seeking at T1, there was a pre-post increase (n = 72, pre: 0.9 ± 0.9; post 1.0 ± 0.5; 95% CI of difference 0.1 - 0.3, p = 0.003). Among participants who reported any use of escape-avoidance at T1 (score > 0), there was a pre-post decrease in this mode of coping (n = 135, pre: 0.7 ± SD 0.5; post 0.6 ± 0.5; 95% CI of difference -0.1 - 0.0, p = 0.01).

Comparing the outcomes among the three doses of the Pandemic Influenza Stress Vaccine (Table 4) reveals that each course length resulted in improved self-efficacy and confidence in training and support. Total interpersonal problems decreased for participants of the medium and long courses but not for those who took the short course. Although there were trends toward a decrease in improvement in self-efficacy with increased course length, and an increase in improvement in interpersonal problems with increased course length, between-group differences in change scores were not statistically significant.

**Table 4:** Course outcomes among three lengths of the Pandemic Influenza Stress Vaccine.

						Pre-Post Difference			
						
		Time				95% Confidence Interval			
						
		Time 1		Time 2					
**Variable**	**Course Length**	**Mean**	**SD**	**Mean**	**SD**	**Mean**	**Lower**	**Upper**	**P**

Pandemic Self-Efficacy	Short	88.4	13.2	95.6	12.8	7.3	4.5	10.0	<0.001
	Medium	86.2	13.7	91.7	14.4	5.4	2.9	8.1	<0.001
	Long	88.7	10.9	91.4	11.0	2.8	0.5	5.0	0.02
									
Confidence in Support and Training	Short	32.2	4.7	33.5	4.0	1.2	0.2	2.3	0.02
	Medium	32.8	5.5	34.0	5.3	1.2	0.3	2.1	0.01
	Long	32.8	4.4	33.8	4.7	1.0	0.4	1.6	0.001
									
Total Interpersonal Problems	Short	31.9	17.4	29.6	17.0	-2.3	-4.8	0.3	0.08
	Medium	31.7	17.0	27.6	16.5	-4.0	-6.2	-1.8	0.001
	Long	30.6	13.5	25.7	13.1	-4.9	-7.6	-2.3	<0.001

## Discussion and Conclusions

This study suggests that interactive computer-assisted training for healthcare workers is feasible and may facilitate improvement in psychological variables that predict resilience to the stress of an outbreak of an emerging infectious disease. Improvements were obtained in three of the four targeted domains of psychological function: pandemic self-efficacy, confidence in support and training, and interpersonal problems.

Improvements in interpersonal problems were similar to those achieved with brief dynamic psychotherapy, in which improvements in socially avoidant, nonassertive and overly accommodating problems are more readily achieved than improvements in the cold, self-centered and controlling dimensions [46]. In this study, there were specific improvements for all participants in most dimensions of interpersonal problems. For problems of being cold or distant there was an improvement for those who reported any such problem at the start of the course. We attribute this success to two factors. First, developing skills to respond to interpersonal difficulties was an explicit focus of the course. Second, participants differ from psychotherapy patients in that they are not selected for having psychiatric disorder and, in fact, report mild interpersonal problems.

Coping by the use of problem-solving, seeking support and escape-avoidance did not change over the course of the Pandemic Influenza Stress Vaccine. This may be because the use of coping strategies can be context-dependent. Since the specific stressful events that require coping at T1 and T2 may differ, contextual changes may affect coping more than changes that result from training. On the other hand, the lack of change in these variables may be because prior to training most participants tended to use problem-solving and support seeking frequently and escape-avoidance infrequently, and thus there was only room for improvement in a subset of the sample. Among those who report under-utilizing support-seeking or problem solving or over-utilizing escape-avoidance at T1, the training was associated with an improvement.

There was a substantial attrition of about 40% of healthcare workers who consented to participate in the course but did not take the training. Since these people did not commence taking the course, the attrition is not attributable to their reactions the course itself, and may rather represent an insufficiently considered intention to take the training, obstacles to finding time (such as high competing demands for healthcare workers' time) or a lack of motivation (due to low perceived salience of pandemic stress prior to an actual pandemic or other factors). Since there was no follow-up with drop-outs in this study to determine reasons for attrition, future research is required to identify barriers to learning and the extent of perceived need for resilience training, especially in nonprofessional hospital staff for whom drop-out rates were highest. Empirically demonstrated efficacy would likely bolster the appeal of this training. Once healthcare workers commence the course, dropping out is less common (20%).

Comparison of different doses of the course suggest that the medium length course is sufficient for significant improvements in pandemic self-efficacy, confidence in support and training, and interpersonal problems. Although there was a trend toward larger improvements in interpersonal problems in the long course, there was also a trend toward a higher drop-out rate. The lack of a statistically significant difference in these measures between groups may indicate that the groups are truly similar or may reflect a lack of power. For example, it would require two groups of 182 participants each to confirm the null hypothesis for a between-group difference in the change in interpersonal problems of the magnitude that we found between the short and long course. To the extent that differences between courses can be detected, and following the principle that the shortest course that meets the training goals is the most desirable, the evidence supports the medium length course as the optimal duration.

Although statistically significant benefits were consistently found, the practical significance of these improvements is untested. Bearing in mind that we measured the mediators of stress-outcomes rather than direct measures of pandemic stress, this study leaves unanswered the question of whether modest improvements in self-efficacy, confidence in support and training, and interpersonal problems are sufficient to reduce pandemic-related stress and absenteeism due to stress. A test of this mode of training which directly measures stress and absenteeism under real-world pandemic conditions could answer this question.

This course mixes several modalities of teaching. While using mixed modalities is consistent with best practices in continuing professional education [47], it does not allow us to identify which component, if any, is the "effective ingredient" of the Pandemic Influenza Stress Vaccine. In particular, it would be useful to test the specific impact of the reflective exercises, both because this mode of teaching is innovative and because these segments are by far the most resource-intensive to design and implement, and are highly specific to the context of the training.

The conclusions of this study are limited by weaknesses in its design. First there was no comparison between computer-assisted learning and face-to-face educational techniques and so this study provides no information about the relative benefits of these approaches. Second, there was no long-term follow up to determine the stability of the benefits that were found. Third, it was only possible to measure proximal predictors of resilience rather than the participants' actual responses to an influenza pandemic. Further research would benefit from attending to these unanswered questions.

These results suggest that further testing of the Pandemic Influenza Stress Vaccine is warranted. The H1N1 influenza pandemic provides both motivation and opportunity for a randomized controlled trial (RCT) of this training under real-world pandemic conditions. An RCT is now underway in which a modified version of the medium length course is compared to (i) a knowledge-based course with similar content and duration but without the reflective interactive exercises and (ii) a waiting condition. This trial will allow testing of the hypothesis that reflective exercises are necessary for the course's benefits and will allow for longer term (6 month) follow-up. The RCT also expands the outcomes measured to include absenteeism, perceived personal risk and subjective stress. Through this RCT, participating in the Pandemic Influenza Stress Vaccine is available free of charge to any English-speaking hospital-based healthcare worker in the world http://www.msh-healthyminds.com/stressvaccine.

Maintaining the well being of health care workers is essential in sustaining the systems in which care is provided. It is also humane, decent and mirrors core values of health care. We expect that resilience training even independent of a influenza pandemic warrants attention to the development and evaluation of interventions.

## Competing interests

The authors declare that they have no competing interests.

## Authors' contributions

RGM conceived of the study, performed the analysis and drafted the manuscript. WJL conceived of the study, authored the interactive training and participated in the analysis. RM produced the reflective videos and coordinated the study. LV, NP, JJH and ML participated in the design of the study and helped to draft the manuscript. MAB consulted on principles of continuing professional education and helped to draft the manuscript. All authors read and approved the final manuscript.

## Pre-publication history

The pre-publication history for this paper can be accessed here:

http://www.biomedcentral.com/1472-6963/10/72/prepub

## Supplementary Material

Additional file 1 **Example of interactive reflective exercise video**. Reflective exercises take < 4 min to complete in addition to the time spent viewing the video. The path through the exercise is highly personalized with more than 3000 possible pathways within the program's logic. The goal is not prescriptive, but rather to increase reflection, diminish non-reflective reactive responses to stress and to increase awareness of multiple possible responses to particular situations. The first few steps of one possible pathway is illustrated below. Q: What is your initial reaction? A: It makes me angry. Q: In what way does this make you angry? A: I deserve more respect. Q: You have answered, "I deserve more respect". How can you make this difficult situation work? A: I will be clear and open in communicating with others. Q: You recognize the importance of clear communication in this situation. What can you do to facilitate effective communication? Etc. (the reflective exercise continues). Click here for file
